# Utilization of Lean & Six Sigma quality initiatives in Indian healthcare sector

**DOI:** 10.1371/journal.pone.0261747

**Published:** 2021-12-23

**Authors:** Gaurav Suman, Deo Raj Prajapati

**Affiliations:** Department of Mechanical Engineering, Punjab Engineering College (Deemed to be University), Chandigarh, India; International Medical University, MALAYSIA

## Abstract

**Purpose:**

The purpose of this paper is to investigate the utilization of Lean & Six Sigma quality initiatives in healthcare sector in India.

**Methodology:**

The survey questionnaires were sent to 454 hospitals through registered postal in all the states of India. The survey questionnaire was designed to assess different quality initiatives; currently implemented in Indian hospitals, factors align with organization’s objectives, reasons for not implementing Lean & Six Sigma and contribution of Lean & Six Sigma projects in healthcare improvement projects etc. A separate section in the questionnaire provides the feedback on implementation of Lean & Six Sigma in various hospitals. The relationships between Lean & Six Sigma and healthcare performance have also been established in this paper.

**Findings:**

It is found that 15 Nos. of hospitals have implemented the Lean tools while 14 Nos. have implemented the Six Sigma tools out of 109 collected responses. This shows the utilization of Lean & Six Sigma in Indian healthcare sector. The ‘Lack of knowledge’ and ‘Availability of resources’ are the major reasons for not implementing Lean & Six Sigma. It is also observed that 22% running projects were related to Lean & Six Sigma out of various improvement projects running in various hospitals.

**Originality:**

There is lack of evidences of similar studies that determines the utilization of Lean & Six Sigma in Indian healthcare sector at the national level. This paper will provide important breakthrough to academicians and healthcare practitioners, who are involved in Lean & Six Sigma research.

**Social implications:**

The present study will create awareness among healthcare practitioners across India for utilization of quality tools that will provide direct benefits to the society.

## 1. Introduction

The concept of quality has been effectively addressed by various manufacturing organizations all over the world. Healthcare is one of the major beneficiary, under the service sector as far as the implementation of quality tools is concerned. Quality in healthcare has always posed a considerable challenge, as the life of the patients is dependent upon the quality provided by the healthcare services. Lean & Six Sigma, as quality initiatives are widely applied in healthcare throughout the globe. However, their application in Indian healthcare sector is still very limited [1]. Annual seminars organised by healthcare federation of India reported the following facts:

- India is accounted for 16% worldly maternal deaths and 27% of neonatal deaths.
- The 22% of global tuberculosis incidences are reported in India.
- Non-communicable diseases accounts for 60% of deaths in India.

The above stated reports demand a thorough survey of hospitals throughout India in order to investigate the utilization of quality initiatives, factors align with organization’s objectives, reasons for not implementing Lean & Six Sigma etc.

Many researchers investigated the utilization of Lean & Six Sigma in their respective countries. Antony and Desai [2] assessed the status of Six Sigma implementation in Indian industries. They investigated the mostly and least used tools, critical success factors, reasons for application of Six Sigma in Indian industries, etc. Antony and Kumar [3] investigated the status of Lean & Six Sigma implementation in Scotland. In addition, they determined benefits of Lean & Six Sigma, success factors and barriers of implementing these quality initiatives in healthcare organizations.

Albliwi et al. [4] assessed the status of Lean Six Sigma implementation in Saudi Arabian hospitals. They found that its utilization was still in its nascent stages in those organizations. Ahmed et al. [5] measured the Lean Six Sigma implementation and quality performance in Malaysian hospitals. The results showed that there were significant differences between the staff of the public hospitals and private hospitals on Lean and Six Sigma initiatives. Peimbert-Garcia et al. [6] assessed the status of Lean & Six Sigma practices in healthcare in Mexico. They observed that only 16% of the respondents had implemented the Lean & Six Sigma projects.

Although many studies based on Lean and Six Sigma survey in healthcare organizations are available, there is lack of evidences of studies that evaluate the Lean & Six Sigma utilization in Indian healthcare sector at the national level. The present paper shows the results of a national level survey conducted in India in relation to the Lean and Six Sigma program implementation in the healthcare sector. The survey evaluates the current quality initiatives, reasons for not implementing Lean & Six Sigma, contribution of Lean & Six Sigma projects in healthcare improvement projects, etc. The relationships between Lean & Six Sigma and healthcare performances are also evaluated and presented in this paper.

Section 2 presents the details of review of literature on the application of Lean and Six Sigma in the healthcare industry, whereas the details of research methodology are discussed in Section 3. The analysis of the survey results is presented in Section 4 while Section 5 discusses the results of the study. The practical implications are explained in Section 6 and the limitations taken up in Section 7. The conclusions drawn and the agenda for future research are presented in Section 8.

## 2. Lean & Six Sigma in healthcare

This section presents the detailed literature reviews on the applications of Lean & Six Sigma in healthcare sector. The various researchers have applied these quality initiatives in healthcare sector to reduce processing time, to increase the productivity and to minimise the variations.

Heuvel et al. [7] considered the length of stay (LOS) as critical to quality characteristic of the delivery room in a hospital. The authors utilized Lean Six Sigma and observed that the problem of insufficient number of delivery rooms led to prolonged LOS. They suggested new procedure and protocols, which reduced average LOS from 11.9 hours to 3.4 hours. Taner et al. [8] improved the Magnetic Resonance Imaging (MRI) quality by using Six Sigma. They found that the main reason for variations in diagnostic imaging process was due to the inadequately trained observer/ technician. In order to improve the quality of the MRI, they recommended appropriate training to the observers/ technicians.

Bush et al. [9] utilized the Six Sigma Define-Measure-Analyse-Improve-Control (DMAIC) philosophy in the women medical centre clinic. They were able to reduce the waiting time from 38 to 8 days, which resulted in the increment of revenue by 73%. Anguelov et al. [10] improved the patient flow in Emergency department (ED) using standard Lean principles. After two years, they observed an increase in patient volume and patient admissions by 18% and 22% per month respectively, with a downfall in the LOS by 3%. Taner and Sezen [11] chose the turnover intentions of doctor in medical emergency services as potential subject for Six Sigma implementation. They observed that the personal stress level and salary were the root causes for high turnover intensions. The findings of the project led to increase of process yield to 78% and increase in the net annual revenue by $0.84 million.

Mandahawi et al. [12] re-designed the ED with the help of design for Six Sigma and discrete event simulation software. They used ProModel software to develop a discrete event simulation model, which facilitate decision making. As a result, they reduced the waiting time and LOS by 61% and 34% respectively. Niemeijer et al. [13] improved the discharge procedure in trauma nursing department using Lean Six Sigma technique. They observed the reduction in average LOS to 8.5 days from 10.4 days after the implementation of the suggestions made for improvement. Allen et al. [14] implemented the Six Sigma DMAIC approach to enhance the service quality of hospital discharge process. They found that physician preparation was the major bottleneck and the average discharge time was reduced to 2.8 hours from 3.3 hours after removing bottleneck.

Yeh et al. [15] reduced the door to balloon time of acute myocardial infraction process with the help of Lean Six Sigma tools. The door to balloon time of the process was decreased by 58.4%, after implementation of suggested solutions, with cost saving of $ 4.422 million in the emergency medical resources. Southard et al. [16] suggested the enhancement of service quality of outpatient surgical process. They made use of Six Sigma along with discrete event simulation and radio frequency identification (RFID) to attain desired objectives. The project resulted in annual cost saving of $1.93 million and time saving per patient of 1.1 hours.

Bhat and Jnanesh [17] applied the Six Sigma and Lean tools to lower down the turnaround time in health record preparation process. The authors investigated that there was fair amount of in-process inventory due to longer turnaround time. In this project, they reduced the turnaround time of the project to 39 minutes from 52 minutes. Dinesh et al. [18] lowered down the waiting time with the help of Six Sigma methodology in an Indian Cardiology office. They suggested various improvements to reduce the waiting time, i.e. appointment of additional staff to handle telephonic calls, registration form modifications, etc. The Authors also observed the complimentary benefits of shortening waiting time for lab reports.

Bhat et al. [19] applied Lean & Six Sigma in Indian medical college hospital to lessen the cycle time of registration process. They found that the cycle time diminished to 1.5 minutes from 3 minutes and standard deviation to 21.2 seconds from 61 seconds. Bhat and Jnanesh [20] claimed to reduce the cycle time of outpatient department services in Indian rural hospital with the help of Lean Six Sigma tools. They employed various tools to shorten the cycle time of the process, i.e. 5S, Kanban, etc. They were able to reduce critical to quality characteristic time from 4.27 minutes to 1.5 minutes. Sarkar et al. [21] applied the Six Sigma methodology in the medical lab of a hospital and they specially emphasized the control phase importance in DMAIC procedure of Six Sigma. The authors investigated that initially staff took seven days to complete 90% of the medical tests. After execution of suggested solutions and proper control of the process, they completed 98% of medical tests within a period of seven days.

Ramaswamy et al. [22] observed the quality of postoperative visual outcome following cataract surgeries with the help of Lean Six Sigma approach. At the end, they reduced average residual spherical errors to 0.25D from 0.36D. There was additional benefit of cost saving from 11.5 crores to 13.5 crores. Sanders and Karr [23] considered the ED for the implementation of Lean Six Sigma. At the end of project, there was 50% reduction in vials used for testing, which ultimately lowered the turnaround time of the process.

Basta et al. [24] focused on the dispatch time of medical lab reports. They utilized Six Sigma DMIAC approach along with standard Lean principles. They observed that initially 12.3% of reports were dispatched on the day of patient’s visit. This was raised to 90.6% after the implementation of solutions, suggested by the Six Sigma procedure. Zhang et al. [25] investigated the implementation of Lean & Six Sigma for improving logistics operations in Singapore. They found the reasons behind some logistics companies not implementing Lean or Six Sigma and determined the benefits and challenges of implementing Lean and Six Sigma in logistics companies. Gheysari et al. [26] reduced the cancellation of surgery using Lean Six Sigma approach. After implementation, there was significant downfall in cancellation of surgery, i.e. from 31 cases to 12.

Montella et al. [27] claimed to lower down the number of patients affected by sentinel bacterial infections using Lean Six Sigma methodology. The project caused reduction of 20% in the average number of hospitalization days between pre-intervention and control phases. Kutsal et al. [28] improved histopathology laboratory productivity using Six Sigma. The project caused the increase in Six Sigma score from 24% to 68%. Honda et al. [29] showed how the Lean and Six Sigma principles improve the hospital performance by taking 35 case studies. They stated that multidisciplinary team approach along with Six Sigma training is the most crucial for successful implementation of Six Sigma. Alkinaidri and Alsulami [30] improved the delays in referral system of hospital using Lean Six Sigma approach. After the project, a little improvement in the response time of physicians to the referral system was achieved. Molla et al. [31] improved the timeliness of the discharge process of the hospital using Lean Six Sigma approach.

Improta et al. [32] applied the Six Sigma to reduce the pre-operative LOS in the Italian hospital. They analysed two groups of patients before and after the introduction of diagnostic therapeutic-assistance path. They were able to achieve overall 54% reduction in LOS. Ahmed et al. [33] investigated the applications of Six Sigma techniques in Malaysian private hospitals. They found that doctors had better perception regarding; process improvement tools to measure process, leadership to continuous improvement processes, training in process improvement tools, etc.

Al Khamisi et al. [34] developed a knowledge-based system (KBS) to support the implementation of Lean Six Sigma principles. They concluded that the KBS provides an enhanced strategic and operational decision-making hierarchy for achieving a performance benchmark. Antony et al. [35] explored the Six Sigma use in the Norwegian pubic healthcare in order to reduce medication errors. They found that the implementation of Lean & Six Sigma in the Norwegian is still in its infancy.

Mousavi et al. [36] quantified the relationship between Lean implementation & occupational health and safety performance. The results of the study supported the importance of using occupational health safety leading indicators to appropriately measure the impact of lean implementation on workers’ health and safety. Al-Zain et al. [37] implemented the Lean Six Sigma to shorten the waiting time of patients in private hospital of Kuwait. They observed 67% reduction in patient waiting time. Al-Qatawneh et al. [38] proposed a framework to apply Six Sigma in the area of healthcare logistics. They also presented a case study, which showed implementation of the proposed framework at a Jordanian hospital.

Khorasani et al. [39] determined the major healthcare problem domain using Lean supply chain management. They also provided a list of the most common techniques for implementing Lean supply chain management in healthcare. Hanifin and Zielenski [40] described a methodology for implementation and sustainment of continuous quality improvement initiatives through committee structures aimed at reducing medication error rates. Vaishnavi and Suresh [41] identified and analysed the major readiness factors for implementing the Lean Six Sigma in health-care organizations using total interpretive structural modelling technique. They found that customer-oriented and goal management cultures are the key readiness factors for Lean Six Sigma. Clay-Williams et al. [42] determined the relationships between quality management systems, safety culture and leadership and patient outcomes in Australian Emergency Departments.

Hundal et al. [43] investigated how the Lean Six Sigma may help to mitigate the impact of COVID-19 within health care environments. The results reported that personal safety is the primary subject, followed by supporting dimensions of process redesign, and telemedicine. Lee et al. [44] evaluated U.S. hospital culture profiles for better performance in patient safety, patient satisfaction, Six Sigma and Lean implementation. The study suggested that hospitals should employ a more comprehensive approach to enhance performance of hospitals.

## 3. Research methodology

The main aim of the study is to investigate the utilization of Lean & Six Sigma quality initiatives in Indian healthcare sector. In order to derive a better insight into the study, the main objective of the study is further sub-divided into following research questions.

- What is the current utilization of Lean & Six Sigma in Indian healthcare sector?
- What are the reasons for not implementing the Lean & Six Sigma in Indian healthcare sector?
- To investigate the experience and role of healthcare practitioners in the Lean & Six Sigma projects, average duration and usefulness of Lean & Six Sigma projects in Indian healthcare sector.
- To investigate the contribution of Lean & Six Sigma projects in the Indian healthcare improvement projects.
- To determine the relationship between Lean & Six Sigma and healthcare performance in India.

Bearing the research objectives of investigation in mind, a survey questionnaire has been designed. The population identified for the study represents the healthcare organizations of India, which include hospitals, medical centres and institutes. The survey questionnaire is designed to gather the collective views and wisdom of professionals in various healthcare organisations. A panel of healthcare, academic and language experts perform the validation of questionnaire items. Their suggestions and comments have been incorporated in the final version of the questionnaire designed.

Since the present study involves a national level survey, it was not feasible to collect data by conducting interviews in person all over India. Hence, the data from the nearby hospitals were collected through direct personal interviews, while the responses from the far off hospitals were received through the registered postal services. The ethical approval has been granted from Student Research Committee (SRC) of the Department of Mechanical Engineering, Punjab Engineering College (Deemed to be University) to conduct survey across all over India. The prior consent was taken from the respondents of different hospitals through emails/telephonically. Target respondents were selected from various healthcare industries, located in different areas of the country. Questionnaires were addressed to either the Director or Principal or Chief Medical officer of the target organizations.

The proportionate stratified sampling method is used to collect the data from the hospitals in India. In the present study, the population is different hospitals throughout India and this population is finite. The sample size (n) for the finite population with categorical data is calculated using Eq 1.

$$n=\frac{z^{2}pq\;N}{e^{2}\left(N-1\right)+z^{2}pq}$$ (1)

Where Z = Normal distribution value

p = Probability of occurrence (0.5)

q = Probability of non-occurrence (1-p = 0.5)

e = Acceptable margin of error (0.05 for categorical data)

N = Population size

The Z value is normally taken at 5% alpha level for engineering applications. So correspondingly, the value of Z at 0.05 level of significance is 1.96. The acceptable margin of error is 0.05 for categorical data, whereas for continuous data, it is 0.03. The population size is the number of hospitals in India and is 20,306 (Source: https://data.gov.in). Therefore, after putting all the values, the computed sample size is 378. In order to obtain the maximum response from different hospitals and to reduce the possible uncertainties in survey questionnaire mishandling, approximately 20% oversampling has been done and final sample size of 454 was computed.

A total number of 454 survey questionnaires were sent to different hospitals all over India. The addresses of the different healthcare organisations across India were obtained from the internet. As stated earlier, consent is taken from the hospital’s authority through either e-mails or telephonically. The sample size (454) is 2.23% of the total population. So the 2.23% hospitals from each state have been considered for the study, as per proportionate stratified sampling method. The incomplete, damaged and torn questionnaires are excluded from the study. A total number of 109 completed responses have been received from the different hospitals. Although the sample size is comprised of more than 100 responses, it is not considered enough to generalize the results of a national level survey.

The study was conducted from April 2019 to March 2020 in the institute itself. In the initial stage, the management and descriptive analysis of received data are performed in Microsoft Excel-2010. The ANOVA test was also performed in Minitab-17 at 0.05 level of significance, assuming equal variances. The reliability and validity of the data were tested using Cronbach’s alpha and Factor loading respectively in SPSS Statistics-23 software.

## 4. Survey results

A total number of 109 responses have been received out of 454 communicated questionnaires of the survey, which shows response rate of 24%. The response rate, greater than 20% is considered as a good response [45, 46]. Recent studies; published in highly ranked management journals typically feature response rates between 30% and 40% [47–49]. However, in the present study, total 109 complete questionnaires were used for the analysis purpose. Table 1 shows the details of respondents who have completed the questionnaires.

**Table 1:** Details of the respondents of survey questionnaire.

Sr. No.	Job Titles	Frequency
1	Director	16
2	Deputy director	2
3	Principal	14
4	Dean Research	1
5	Registrar	0
6	Manager	18
7	General manager	1
8	Head of department	14
9	Employee/Doctor	23
10	Staff	1
11	Medical Superintendent	13
12	Others	6
	**Total**	**109**

It is clear from Table 1 that all the respondents are working at important medium/high level positions in the hospitals. Most of the respondents are Doctors (23 Nos.), Managers (18 Nos.), Directors (16 Nos.), Principals (14 Nos.), Heads of department (14 Nos.) and Medical Superintendents (13 Nos.). The six respondents have chosen ‘Other’ option under ‘Job Title’ and they mentioned their profile as Chief Medical Officer (CMO), Senior Medical Officer (SMO) and supervisor etc.

Table 2 shows the respondents’ experience in the healthcare organizations. It is clear that majority of respondents (42 Nos.) have an experience of 10 to 20 years, whereas 39 respondents have spent more than 20 years in the healthcare sectors.

**Table 2:** Respondent’s experience in healthcare organizations.

Sr. No.	Experience in healthcare organizations	Frequency of respondents
1	Less than 1 year	2
2	1 to 5 years	8
3	6 to10 years	18
4	10 to 20 years	42
5	More than 20 years	39
	**Total**	**109**

The results of survey questionnaire are discussed in the following subsections.

### 4.1 Utilization of quality initiatives

**Fig 1:**
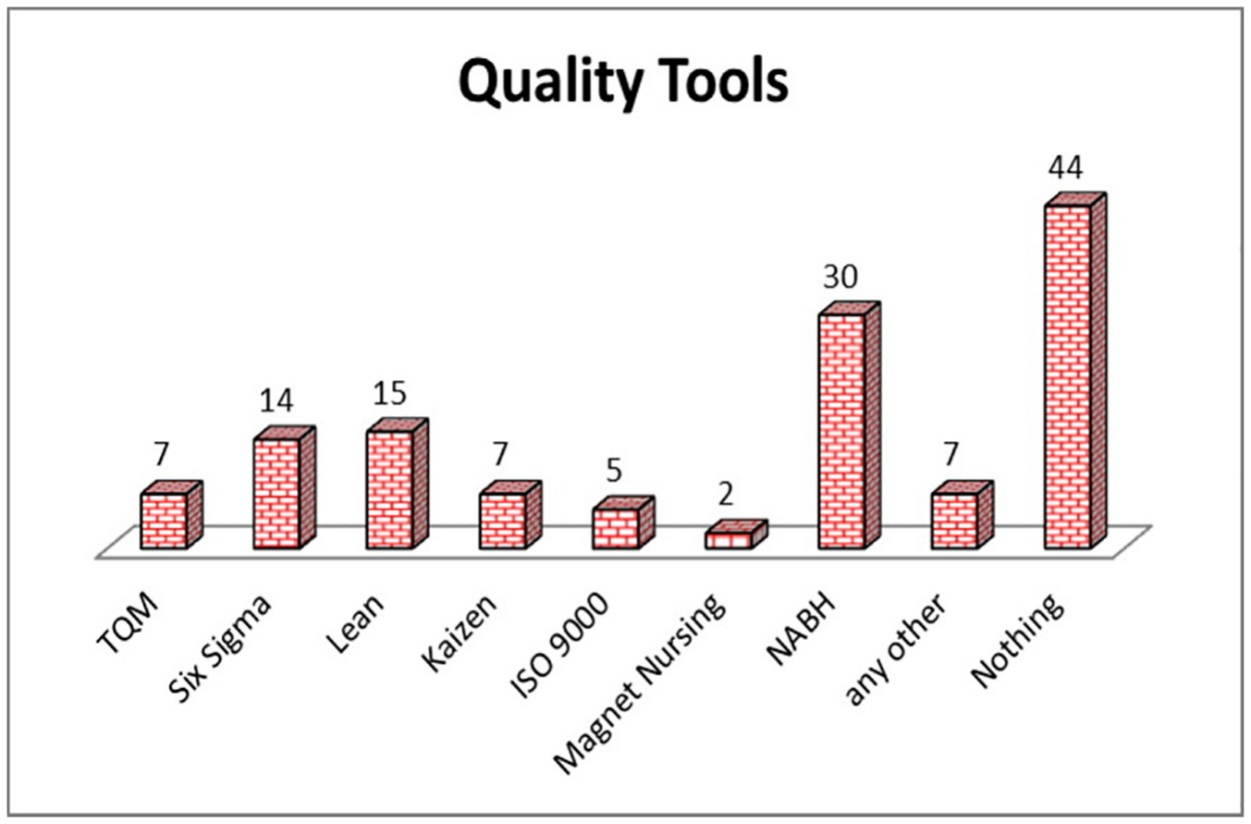
Implementation of various quality tools in different hospitals.

The respondents were asked about the various quality initiatives, implemented in their hospitals. Fig 1 shows the frequency of hospital implemented different quality initiatives. It is clear that more than 40% of the hospitals (44 Nos.) have not implemented any quality tools. The numbers of hospitals, which have implemented Lean & Six Sigma techniques are 15 and 14 respectively. This shows the utilization of Lean & Six Sigma in Indian healthcare sector. The mostly applied quality tool is National Accreditation Board for Hospitals (NABH) and 30 numbers of hospitals have applied the same. Hospitals numbering seven have implemented TQM and Kaizen techniques in their hospitals.

The implementations of Lean & Six Sigma across various regions of India are also assessed. The whole country is divided into four regions, i.e. North, South, East and West regions. The implementation of Lean & Six Sigma is individually assessed for each region. The details of Lean & Six Sigma in different regions are given in Table 3. It is clear form Table 3 that in the Northern region, six states have applied the Lean & Six Sigma eight times. Similarly, the applications of Lean & Six Sigma in other regions are shown in Table 3.

**Table 3:** Detail of Lean & Six Sigma in different regions of India.

Region	Number of states applied Lean & Six Sigma	Frequency of applied Lean & Six Sigma
**North**	6	8
**South**	3	7
**East**	5	6
**West**	1	1

In order to find out whether the differences in the results of the various regions are statistically significant or not, the Analysis of Variance (ANOVA) is used. An ANOVA method is a useful tool to determine whether two or more populations are statistically different from each other. The region is taken as factor at four levels, i.e. North, South, East and West regions. The all means are assumed equal in null hypothesis and it is assumed that at least one mean is different in alternative hypothesis. The analysis is performed at 95% level of confidence and the results are computed from Minitab-17 software and presented in Table 4.

**Table 4:** ANOVA results for different regions.

Source	Degree of freedom	Sum of square	Mean sum of square	F value	p value
**Factor**	3	4.833	1.611	1.90	0.163
**Error**	20	17.000	0.850		
**Total**	23	21.833			

It is clear from the Table 4 that the ‘p’ value is greater than 0.05, which suggests rejection of alternative hypothesis. It shows that, there is no significant difference among the results of various regions in India.

**Fig 2:**
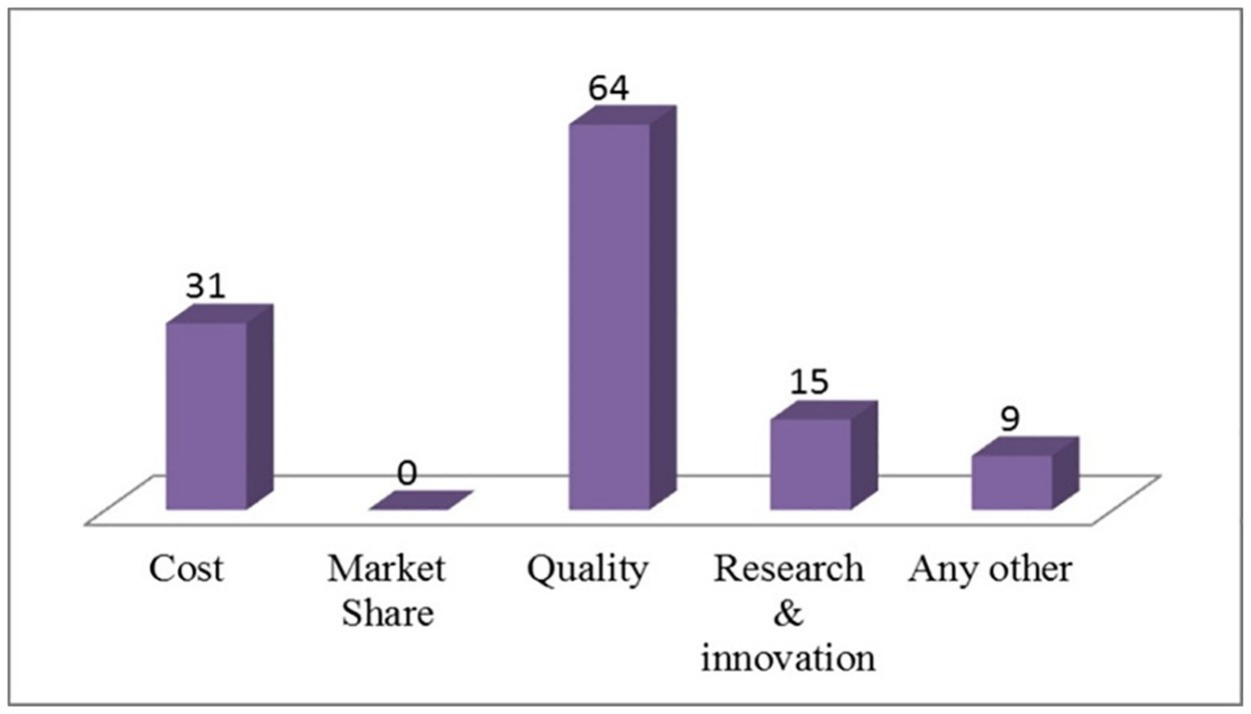
Factors align with organization’s strategic objectives.

The top most critical factors that align with organization’s strategic objectives have been investigated. It is observed from Fig 2 that majority of responses (64 Nos.) are in the favour of quality, which means quality is one of the main criteria during formulation of hospital’s policies and objectives. In addition to that cost is also an important factor and accounts for 26.6% (31 Nos.) weightage. The research & innovation activities account for only 11.9% (15 Nos.), which shows that India is lacking in research-focused hospitals. A point to be noted is that, no participating hospitals have chosen market share as a factor that aligns with the organization’s strategic objectives.

The physical presence of quality department in hospitals is assessed on the basis of the replies from the respondents. It is found that 67.9% (74 Nos.) of the hospitals in India do not have quality department. Overlooking whether or not hospitals implemented Lean or Six Sigma, the respondents were asked questions on their knowledge of Lean and Six Sigma. It is analysed that only 38% respondents have the knowledge of Lean & Six Sigma methodologies.

**Fig 3:**
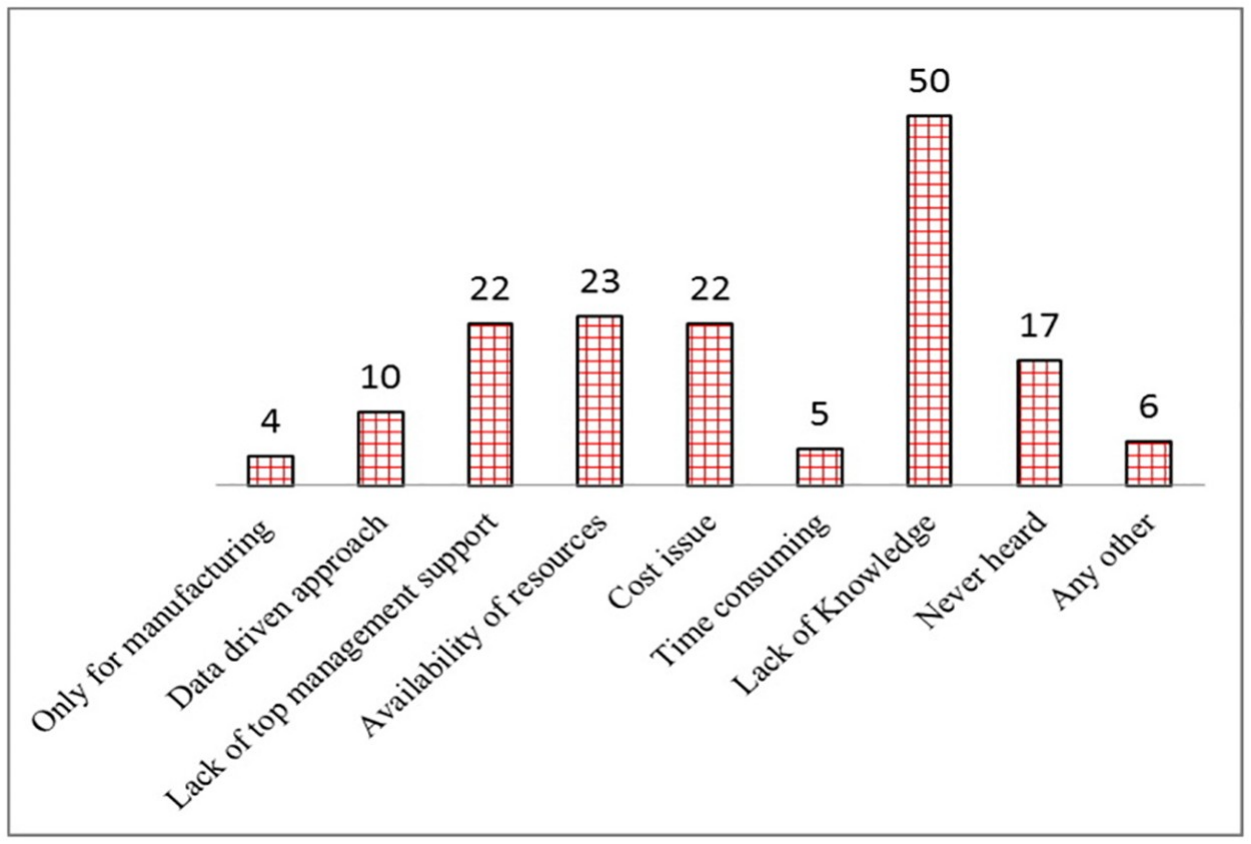
Frequency of the reasons for not implementing Lean & Six Sigma.

The respondents were also asked the specific questions regarding Lean & Six Sigma, i.e. ‘whether their hospitals implemented Lean & Six Sigma’. It is observed that only 6.4% (7 Nos.) have implemented both Lean & Six Sigma. The 79.8% of the hospitals have applied neither Lean nor Six Sigma. The respondents were also asked about their future plan, i.e. ‘whether they will implement Lean & Six Sigma in future’ and it is found that 70.6% (77 Nos.) of the respondents are agreed upon applying Lean & Six Sigma in future in their hospitals. Questions to understand the factors underlying non-implementation of Lean or Six Sigma in the Indian hospitals were also asked from the respondents and Fig 3 shows the results.

It is found that majority of responses (50 Nos.) are in the favour of ‘Lack of knowledge’ for not implementing Lean & Six Sigma. 14.47% (23 Nos.) of the respondents have pointed out ‘Availability of resources’ as the reason for the same. After that, equal weightages (22 Nos.) are given to the ‘Lack of top management support’ and ‘Cost issue’. About 10.7% (17 Nos.) of respondents have marked the ‘Never heard’ option. Approximately; 3.8% (6 Nos.) of respondents have chosen ‘Any other’ option and they wrote ‘No need’, ‘Lack of manpower’, etc. as reasons for not implementing Lean & Six Sigma.

### 4.2 Questionnaire for Lean or Six Sigma organizations

**Fig 4:**
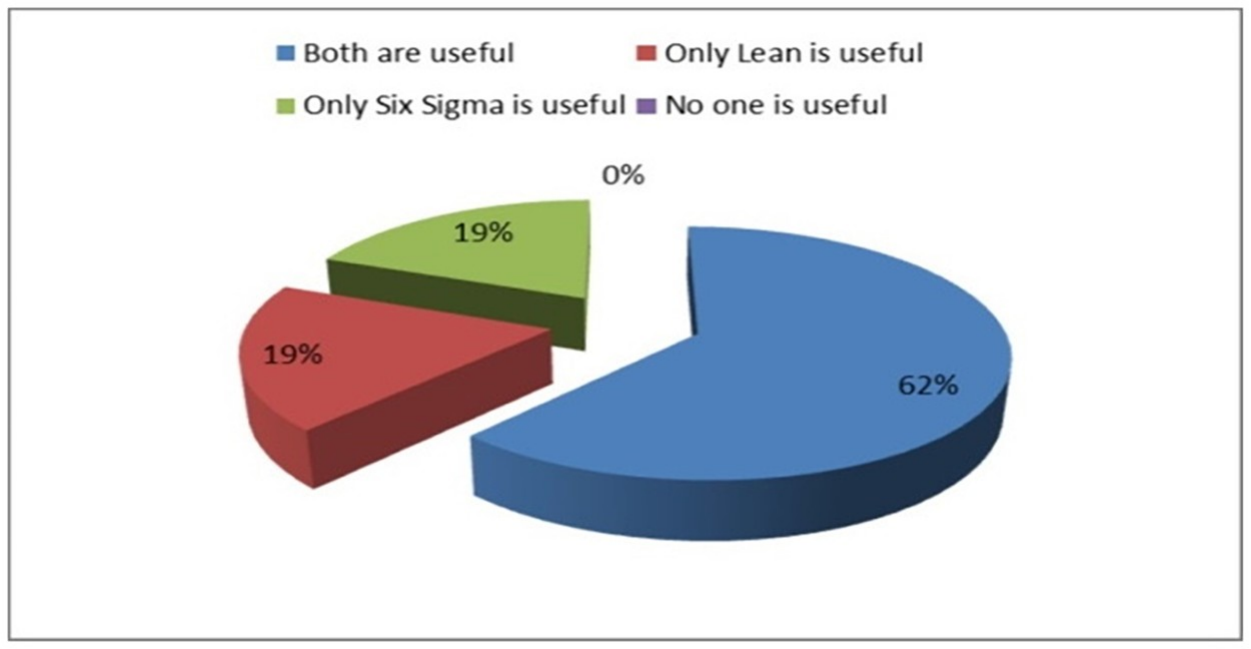
Pie chart showing the usefulness of Lean & Six Sigma.

The specific questions were put only before those hospitals, which had applied Lean or Six Sigma. Fig 4 shows the result of usefulness of Lean & Six Sigma received from the hospitals, which have implemented it. It is clear from the Fig 4 that 62% (13 Nos.) of the hospitals found both Lean & Six Sigma useful, whereas 19% of them individually found Lean and Six Sigma to be beneficial.

A question regarding the role of respondents in the Six Sigma and Lean projects in hospitals was also addressed. It is found that five respondents are not involved in the projects. The seven respondents are working as Senior Managers and five respondents are working as Six Sigma leaders in the Lean & Six Sigma projects, whereas one respondent was green belt. Three respondents have chosen the option of ‘Any other’ and working as Administrator, Nodal officer and Project Champion.

The experience of respondents with Lean & Six Sigma was also assessed through survey questionnaire. Table 5 shows the experience of respondents with Lean & Six Sigma. It is clear that in case of Six Sigma, nine respondents have no experience, whereas only one respondent is having the experience of more than 5 years. The seven respondents have an experience of less than 2 years and four respondents are working with the experience of 2 to 5 years. Similarly, nine respondents have no experience with Lean, whereas only one respondent has shown an experience of more than 5 years. The nine respondents have less than 2 years of experience and two respondents have an experience of 2 to 5 years of Lean techniques.

**Table 5:** Frequency of respondents having experience with Lean & Six Sigma.

Sr. No.	Experience	With Six Sigma	With Lean
1	None	9	9
2	Less than 2 years	7	9
3	2–5 Years	4	2
4	More than 5 years	1	1

The average duration of Lean & Six Sigma projects is assessed through survey questionnaire. It is found that most of the projects (9) have average duration of less than 6 months. After that, 8 projects have been completed between 6 months to one year. The duration of three projects was more than 2 years and one project has the duration of between one and two years.

### 4.3 Contribution of Lean & Six Sigma initiatives in healthcare improvement projects

The contribution of Lean & Six Sigma projects in hospital’s improvement in India is also assessed. To find the information on contribution, number of Lean, Six Sigma and Lean Six Sigma projects completed or still running in the hospitals were also assessed. Along with that, total number of improvement projects, other than Lean & Six Sigma were also assessed through the survey questionnaire. The 52 number of hospitals have implemented at least one type of quality initiatives including Lean & Six Sigma. Table 1A (S1 Appendix) shows the contribution of Lean & Six Sigma projects in healthcare improvement initiatives. It is clear from the Table 1A in S1 Appendix that average contribution of Lean & Six Sigma in healthcare improvement projects in the hospitals is 21.32%.

### 4.4 Overall performance of Indian hospitals

The overall performance of hospitals is divided into two categories, i.e. ‘Quality performance’ and ‘Business performance’. The quality and business performance measures are independently assessed from the collected data of various hospitals. The questions were assessed on five point Likert scale; where one represents ‘strongly disagree’ and five shows ‘strongly agree’. The questionnaire to assess quality and business performance is given in Table 1B (S2 Appendix). The average quality performance of the hospitals is computed to be 3.44, whereas, the mean business performance comes out to be 3.05. The overall hospital performance is the average of quality and business performance. Therefore, the overall performance of all the participating hospitals is 3.25 and considered as the average performance of hospitals in India.

Reliability and Validity tests of collected data are performed using SPSS Statistics-23 software for quality and business performance of the hospitals. Table 6 shows the reliability test for the performance of hospitals. It is clear that Item 1 to Item 7 (Table 1B –S2 Appendix) are quality performance questions and from Item 8 to Item 13 are business performance questions. Before performing reliability test, corrected item minus total correlation (CIMTC) is calculated. CIMTC is the correlation coefficient between individual item and remaining items. Items having a relatively low correlation (≤ 0.30) with the other items have to be deleted prior to further analysis [50]. Table 6 clearly shows that the value of CIMTC is much higher than 0.30 for quality performance as well as for business performance of the hospitals, which suggest to proceed for reliability test; i.e. Cronbach’s alpha (α). The value of Cronbach’s alpha (α) is computed as 0.908 and 0.832 for quality and business performance of hospitals respectively. The value of alpha (α) greater than 0.70 is generally considered good and proves that the collected data are highly reliable [51]. The last column of Table 6 shows the value of alpha (α), when the particular item is deleted. It is clear that value of alpha (α) decreases when the specific item is deleted in quality and business performance of the hospitals. This represents that each item has positive contribution towards quality as well as business performance of the hospitals. A sample calculation for Cronbach’s alpha is given in S3 Appendix.

**Table 6:** Reliability testing for performance of hospitals.

Sr. No.	Performance	Factors	CIMTC	Cronbach alpha (α)	α, if Item Deleted
1	Quality Performance	Item 1	0.711	0.908	0.896
2		Item 2	0.626		0.905
3		Item 3	0.706		0.897
4		Item 4	0.751		0.892
5		Item 5	0.788		0.888
6		Item 6	0.720		0.896
7		Item 7	0.788		0.888
8	Business Performance	Item 8	0.540	0.832	0.817
9		Item 9	0.654		0.794
10		Item 10	0.686		0.787
11		Item 11	0.750		0.773
12		Item 12	0.475		0.830
13		Item 13	0.530		0.821

Table 7 shows the factor loading for the performance of hospitals. Before conducting validity test, the Kaiser-Meyer-Olkin (KMO) test is performed to check; whether distribution of values or sample size is adequate for conducting factor analysis. Kaiser himself suggested the value of KMO greater than 0.50 as acceptable [52]. Factor loading is a method used for validity analysis. Factor loadings of ± 0.50 or greater are considered practically significant [51]. It is clear from Table 7 that KMO values are significantly higher than 0.50 for both, quality and business performance measures of hospitals, and it shows that adequate sample size is taken for conducting the factor analysis. The values of factor loading are also much higher than 0.50 for all the items and that confirm that the collected data are valid. A sample calculation for Factor Loading is given in S3 Appendix.

**Table 7:** Factor loading for performance of hospitals.

Sr. No.	Performance	Factors	Factor Loading	KMO
1	Quality Performance	Item 1	0.791	0.922
2		Item 2	0.718	
3		Item 3	0.788	
4		Item 4	0.823	
5		Item 5	0.855	
6		Item 6	0.798	
7		Item 7	0.85	
8	Business Performance	Item 8	0.857	0.782
9		Item 9	0.806	
10		Item 10	0.826	
11		Item 11	0.884	
12		Item 12	0.893	
13		Item 13	0.873	

### 4.5 Relationship between Lean & Six Sigma and healthcare performance

In order to find the relationship between Lean & Six Sigma and performance of hospitals, the performances of hospitals are evaluated with and without the use of Lean or Six Sigma. So, the hospitals; implemented Lean or Six Sigma are analysed separately from hospitals which have not implemented the same. The mean scores of items 1 to 7 (Table 1B –S2 Appendix) for quality performance, with and without Lean or Six Sigma have been calculated from the received responses are shown in Table 8.

**Table 8:** Mean scores of items 1 to 7 for quality performance.

	Item 1	Item 2	Item 3	Item 4	Item 5	Item 6	Item 7
**Without Lean or Six Sigma (85)**	**3.29**	**3.14**	**3.42**	**3.25**	**3.21**	**3.48**	**3.07**
**With Lean or Six Sigma (24)**	**3.96**	**3.92**	**4.13**	**3.96**	**4.17**	**4.21**	**4.08**

The ANOVA test is performed to know, whether there is a significant difference between quality performance with and without Lean or Six Sigma. This analysis is performed at 95% confidence level and the results are computed from Minitab-17 software and presented in Table 9.

**Table 9:** ANOVA results for quality performance.

Source	Degree of freedom	Sum of square	Mean sum of square	F value	p value
**Factor**	1	2.21	2.21	128.31	0.00
**Error**	12	0.20	0.01		
**Total**	13	2.42			

It is clear from the Table 9 that the ‘p’ value is less than 0.05 (equals to zero), which suggests the rejection of null hypothesis. It shows that there is a significant difference between quality performance of hospitals; with and without implementation of Lean or Six Sigma.

The mean scores of items 8 to 13 (Table 1B –S2 Appendix) for business performance, with and without Lean or Six Sigma are given in Table 10.

**Table 10:** Mean scores of items 8 to 13 for business performance.

	Item 8	Item 9	Item 10	Item 11	Item 12	Item 13
**Without Lean or Six Sigma (85)**	**3.22**	**3.02**	**2.82**	**2.92**	**2.46**	**2.73**
**With Lean or Six Sigma (24)**	**3.88**	**3.88**	**3.63**	**3.83**	**3.21**	**3.79**

Once again, ANOVA is performed at 95% confidence level with the help of Minitab-17 software and results presented in Table 11. It is found from Table 11 that ‘p’ value is much smaller than 0.05, which means that there is a significant difference between business performance with and without implementation of Lean or Six Sigma.

**Table 11:** ANOVA results for business performance.

Source	Degree of freedom	Sum of square	Mean sum of square	F value	p value
**Factor**	1	2.12	2.12	31.62	0.00
**Error**	10	0.67	0.06		
**Total**	11	2.79			

Table 12 shows the mean quality and business performance of hospitals with and without Lean or Six Sigma. It is clear that quality performance is better for those hospitals, which have implemented Lean or Six Sigma. Similar pattern can be observed for business performance, where performance is increasing due to implementation of Lean or Six Sigma. It is found that average quality performance is 4.06 for those hospitals; using Lean or Six Sigma, whereas performance has been degraded to 3.27 for the hospitals, which did not apply the same. Similarly, business performance is 3.70 for the hospitals using Lean or Six Sigma and it is 2.86 without application of Lean and Six Sigma.

**Table 12:** Comparison of performance of hospitals with and without Lean or Six Sigma.

	Mean Quality Performance	Mean Business Performance	Overall Performance
**Without Lean or Six Sigma (85)**	3.27	2.86	3.08
**With Lean or Six Sigma (24)**	4.06	3.70	3.89

The performances of hospitals are also evaluated with and without any quality initiatives. Table 13 shows the quality and business performance of hospitals with and without quality initiatives. It can be observed that quality performance is more (3.77) when the hospitals have implemented some type of quality initiatives; and it is less (3.15) when the hospitals have not implemented any quality initiatives. It is also found that the overall performance of hospitals is low for organizations, which did not implement any quality tools.

**Table 13:** Comparison of performance of hospitals with and without quality initiatives.

	Mean Quality Performance	Mean Business Performance	Overall Performance
**Without Quality initiatives (57)**	3.15	2.78	2.98
**With Quality initiatives (52)**	3.77	3.34	3.57

## 5. Discussions

The present study is essentially a national level survey in India to find the current quality initiatives practices in the healthcare sector. The hospitals in different states and union territories of India are the population for the study. Pre-testing of the questionnaire has done by the panel of academicians and healthcare experts to ensure the clarity of the questions. Out of 454 surveys communicated, 109 responses were received, which may be considered as good response rate [45]. The questionnaires were filled in by experienced authorities of the hospitals, employed against high-level designations, such as Director, Principal, Head of Department, Medical Superintendent, Manager, etc.

The respondents were asked for the current quality initiatives practices in their hospitals. It is observed that nearly 40% of the hospitals did not apply any type of quality initiatives. The survey results show that there are only 15 hospitals that applied Lean technique while, 14 hospitals have implemented Six Sigma. It shows that application of Lean & Six Sigma is still in its early stages in India. Although Lean & Six Sigma are rapidly expanding in healthcare sector throughout the globe, its applications are still limited in Indian healthcare sector [1]. Albliwi et al. [4] assessed the status of Lean Six Sigma implementation in developing country i.e. Saudi Arabian organisations. They also found similar results as the implementation status still in its growing stage. This represents that strategies like Lean and Six Sigma has received less attention in developing countries.

Most of the respondents have given reasons such as ‘Lack of Knowledge’ for not implementing Lean or Six Sigma. ‘Availability of Resources’ is the other major reason for not applying the methodology. Antony and Desai [2] also investigated the ‘Lack of awareness’ and ‘Satisfaction with other quality improvement initiatives’ as the main reasons for not implementing Six Sigma program in Indian manufacturing and service industries. It is found that only 38% of the respondents have the basic knowledge of Lean and almost similar results are observed for Six Sigma. This proves that proper training and knowledge should be provided to the healthcare practitioners by the top-level management about different quality initiatives like Lean & Six Sigma to facilitate quality and safety in the hospitals. It is also observed from the results of the survey that, some of the healthcare professionals have rigid mind-set, as approximately 30% of the respondents didn’t agree on applying Lean & Six Sigma in their hospitals in near future.

Another surprising fact observed from the survey questionnaire is that, only 32% of the hospitals have the Quality department. While drafting and designing a hospital’s policies and strategies, it needs to be acknowledged that attention to quality is one of the most critical factors in the Indian healthcare sector. This establishes that healthcare professionals in India are aware of the importance of quality in hospitals. Yeh et al. [15] and Southard et al. [16] also emphasized the importance of quality to increase the satisfaction level of customers. Cost is yet another factor, which is considered important with respect to hospital policies. None of the respondents have selected ‘Market share’ as a factor that aligns with organization’s strategic objectives. This shows that hospitals’ policies and objectives are basically customer focused and irrespective of business and market share. The contribution of Lean & Six Sigma projects in the hospital’s improvement project is assessed through survey questionnaire. The contribution is computed to be 21.32%, which means out of 100, approximately 22 projects belong to Lean & Six Sigma in particular Indian hospitals. The above figure is quite low as the Lean & Six Sigma has not been expanded to its full potential in India.

The relationship between Lean & Six Sigma and healthcare performance of hospitals is also assessed from the data received from the respondents. Survey questionnaire on five point Likert scale is used as given in Table 1B (S2 Appendix). It is found that though number of healthcare organizations with Lean or Six Sigma is very less in India, there is a significant difference between performances of both types of the organizations. The better performance of those hospitals is observed, which apply either Lean or Six Sigma tools. Similarly, performance of hospitals is evaluated with and without the application of quality initiatives. It is observed that the organization which applied any quality tools have better performance than others. This represents the importance of quality initiatives in the healthcare sector in India. Sarkar et al. [21], Ramaswamy et al. [22] and Montella et al. [27] also emphasised that healthcare quality can be improved with the use of Lean and/or Six Sigma implementation.

## 6. Practical implications

Since the implementation of Lean & Six Sigma in India is in its initial stage, this study is an attempt to generate awareness among the Indian healthcare professionals about proven quality tools, i.e. Lean & Six Sigma. The study findings will be very much beneficial to the healthcare professionals, Lean & Six Sigma practitioners and researchers in India. This paper provides insights for researchers, managers and decision makers in healthcare sector. This study stresses upon the relationship of Lean & Six Sigma with healthcare performance, contribution of Lean & Six Sigma projects as healthcare improvement projects, factors align with organization’s strategic objectives, etc. Implementing Lean & Six Sigma in Indian healthcare sector can help organizations to increase service quality, patient satisfaction and productivity by reducing processing time, errors, complications and variations. It is expected that the results of the present study will promote the application of Lean & Six Sigma in Indian healthcare sector.

## 7. Limitations

There are some inherent limitations involved in this study. Firstly, the sample size is taken moderate due to time and budget constraints. Second limitation was the accuracy of the addresses of the organizations or name of concerned persons, as these addresses were taken from the internet. As the data were collected through postal survey, therefore, it is difficult to get deeper insights picture from the survey results. Although, the response rate is 24% (109 Nos.), it is not enough for generalizing the results for national level survey. It is expected that present study will attract more Indian healthcare organizations to participate in the future studies. Another limitation of survey study is that respondents might not reveal the correct status of their organizations. Since respondents are not interviewed directly, there are chances of misunderstanding of some questions and their perspective/purpose.

## 8. Conclusions and agenda for future research

The present paper assesses the different quality initiatives; currently in practice in Indian healthcare sector. It is concluded that implementation of Lean & Six Sigma in India is still in its growing phase. The implementation of Lean and Six Sigma is also assessed across various regions of India through ANOVA and it is found that there is no significant difference among the results of various regions in India. The ‘Lack of knowledge’ and ‘Availability of resources’ are the major reasons for not implementing Lean & Six Sigma. It is also observed that out of 100 improvement projects running in the hospitals; only 22 running projects are related to Lean & Six Sigma. The proper training and knowledge should be provided to healthcare practitioners/management about different quality initiatives available. It is also found that performances of Indian hospitals have strong positive relation with quality initiatives implementation, i.e. Performance is greater for those hospitals, which have applied any quality initiatives.

As a part of future research, study can be performed in a longitudinal way with large sample size. In order to get a deeper insight picture of the results, one can pursue for semi-structured interviews and more qualitative questions can be mixed with quantitative ones in survey questionnaire. Since Lean & Six Sigma quality tools are new to Indian healthcare practitioners and management, one can investigate the drivers and challenges for smooth and effective implementation of Lean & Six Sigma in future.

## Supporting information

S1 Appendix (DOCX)

S2 Appendix (DOCX)

S3 Appendix (DOCX)
