# Supplementary material for: Utilization of Lean & Six Sigma quality initiatives in Indian healthcare sector
Source: PLoS One. 2021 Dec 23;16(12):e0261747. doi: 10.1371/journal.pone.0261747 (PMC8699985; doi:10.1371/journal.pone.0261747)
Supplement: S1 Appendix — (DOCX) [file pone.0261747.s001.docx]

**Appendix: A**

**Table 1A.** **Contribution of Lean & Six Sigma projects in healthcare improvement projects.**

| Sr. No. | Six Sigma Projects | Lean Projects | Lean Six Sigma Projects | Total  (Lean and Six Sigma) | Healthcare Improvement Project (Except L&SS) | Grand Total | %age of Lean and Six Sigma projects |
| --- | --- | --- | --- | --- | --- | --- | --- |
|  | 0 | 0 | 0 | 0 | 3 | 3 | 0 |
|  | 0 | 0 | 0 | 0 | 5 | 5 | 0 |
|  | 0 | 0 | 0 | 0 | 6 | 6 | 0 |
|  | 0 | 0 | 0 | 0 | 9 | 9 | 0 |
|  | 0 | 0 | 0 | 0 | 2 | 2 | 0 |
|  | 0 | 0 | 0 | 0 | 18 | 18 | 0 |
|  | 0 | 0 | 0 | 0 | 20 | 20 | 0 |
|  | 0 | 0 | 0 | 0 | 1 | 1 | 0 |
|  | 0 | 0 | 0 | 0 | 6 | 6 | 0 |
|  | 0 | 1 | 1 | 2 | 0 | 2 | 100 |
|  | 5 | 1 | 1 | 7 | 35 | 42 | 16.67 |
|  | 4 | 4 | 3 | 11 | 21 | 32 | 34.37 |
|  | 1 | 1 | 0 | 2 | 13 | 15 | 13.33 |
|  | 0 | 0 | 0 | 0 | 7 | 7 | 0 |
|  | 0 | 0 | 0 | 0 | 1 | 1 | 0 |
|  | 0 | 3 | 0 | 3 | 7 | 10 | 30 |
|  | 1 | 1 | 1 | 3 | 1 | 4 | 75 |
|  | 0 | 2 | 0 | 2 | 2 | 4 | 50 |
|  | 1 | 1 | 1 | 3 | 14 | 17 | 17.64 |
|  | 22 | 0 | 0 | 22 | 9 | 31 | 70.96 |
|  | 0 | 3 | 0 | 3 | 1 | 4 | 75 |
|  | 0 | 0 | 0 | 0 | 1 | 1 | 0 |
|  | 0 | 0 | 0 | 0 | 2 | 2 | 0 |
|  | 0 | 0 | 0 | 0 | 14 | 14 | 0 |
|  | 1 | 0 | 1 | 2 | 1 | 3 | 66.67 |
|  | 0 | 1 | 0 | 1 | 0 | 1 | 100 |
|  | 0 | 0 | 0 | 0 | 3 | 3 | 0 |
|  | 0 | 0 | 0 | 0 | 1 | 1 | 0 |
|  | 0 | 1 | 0 | 1 | 5 | 6 | 16.67 |
|  | 0 | 3 | 0 | 3 | 4 | 7 | 42.85 |
|  | 0 | 0 | 0 | 0 | 17 | 17 | 0 |
|  | 0 | 0 | 0 | 0 | 9 | 9 | 0 |
|  | 0 | 0 | 0 | 0 | 9 | 9 | 0 |
|  | 0 | 2 | 0 | 2 | 5 | 7 | 28.57 |
|  | 6 | 11 | 1 | 18 | 4 | 22 | 81.81 |
|  | 5 | 8 | 4 | 17 | 5 | 22 | 77.27 |
|  | 1 | 1 | 0 | 1 | 6 | 7 | 14.28 |
|  | 3 | 4 | 4 | 11 | 17 | 28 | 39.28 |
|  | 0 | 0 | 0 | 0 | 16 | 16 | 0 |
|  | 5 | 0 | 0 | 5 | 13 | 18 | 27.77 |
|  | 0 | 0 | 0 | 0 | 10 | 10 | 0 |
|  | 5 | 0 | 0 | 5 | 14 | 19 | 26.31 |
|  | 0 | 11 | 0 | 11 | 23 | 34 | 32.35 |
|  | 0 | 0 | 0 | 0 | 3 | 3 | 0 |
|  | 0 | 0 | 0 | 0 | 6 | 6 | 0 |
|  | 0 | 0 | 0 | 0 | 6 | 6 | 0 |
|  | 0 | 0 | 0 | 0 | 14 | 14 | 0 |
|  | 8 | 0 | 0 | 8 | 23 | 31 | 25.80 |
|  | 0 | 0 | 0 | 0 | 9 | 9 | 0 |
|  | 8 | 9 | 6 | 23 | 27 | 50 | 46 |
|  | 0 | 0 | 0 | 0 | 3 | 3 | 0 |
|  | 0 | 0 | 0 | 0 | 10 | 10 | 0 |
| **Mean contribution of Lean & Six Sigma projects in healthcare** | | | | | | | **21.32%** |
