# Supplementary material for: Utilization of Lean & Six Sigma quality initiatives in Indian healthcare sector
Source: PLoS One. 2021 Dec 23;16(12):e0261747. doi: 10.1371/journal.pone.0261747 (PMC8699985; doi:10.1371/journal.pone.0261747)
Supplement: S2 Appendix — (DOCX) [file pone.0261747.s002.docx]

**Appendix: B**

Please circle or tick the number to indicate the extent to which you agree or disagree on statements that reflect the **degree of change of performance in your hospital** over the past three years.

**1 = Strongly Disagree, 2 = Disagree, 3 = Neutral, 4 = Agree, 5 = Strongly Agree**

**Table 1B.** **Quality performance and business performance questionnaire.**

| **Quality Performance** | | | | | | |
| --- | --- | --- | --- | --- | --- | --- |
| **Items** | **Factors** | **Rating** | | | | |
| Item 1 | The **Length of Stay** of the patients in our hospital has been **reduced** over the past three years. | 1 | 2 | 3 | 4 | 5 |
| Item 2 | The **Waiting time** of the patients in our hospital has been **reduced** over the past three years. | 1 | 2 | 3 | 4 | 5 |
| Item 3 | The **Safety Measure** for the patients in our hospital has been **improved** over the past three years. | 1 | 2 | 3 | 4 | 5 |
| Item 4 | The **Re-admission rate** of the patients in our hospital has been **reduced** over the past three years. | 1 | 2 | 3 | 4 | 5 |
| Item 5 | The **Mortality rate** of the patients in our hospital has been **reduced** over the past three years. | 1 | 2 | 3 | 4 | 5 |
| Item 6 | The **Satisfaction level** of the patients in our hospital has been **improved** over the past three years. | 1 | 2 | 3 | 4 | 5 |
| Item 7 | The **incidence** in our hospital has been **reduced** over the past three years. | 1 | 2 | 3 | 4 | 5 |
| **Business Performance** | | | | | | |
| Item 8 | The **Average Treatment Charge** of the patients in our hospital has been **reduced** over the past three years. | 1 | 2 | 3 | 4 | 5 |
| Item 9 | The **Claims Denial Rate** in our hospital has been **reduced** over the past three years. | 1 | 2 | 3 | 4 | 5 |
| Item 10 | The **Average Insurance Claim’s Processing Time & Cost** in our hospital has been **reduced** over the past three years. | 1 | 2 | 3 | 4 | 5 |
| Item 11 | The **Average Cost per Discharge** of the patients in our hospital has been **reduced** over the past three years. | 1 | 2 | 3 | 4 | 5 |
| Item 12 | The **Market Share** of our hospital has been **increased** over the past three years. | 1 | 2 | 3 | 4 | 5 |
| Item 13 | The **Revenue** of our hospital has been **increased** over the past three years. | 1 | 2 | 3 | 4 | 5 |
