# Supplementary material for: Utilization of Lean & Six Sigma quality initiatives in Indian healthcare sector
Source: PLoS One. 2021 Dec 23;16(12):e0261747. doi: 10.1371/journal.pone.0261747 (PMC8699985; doi:10.1371/journal.pone.0261747)
Supplement: S3 Appendix — (DOCX) [file pone.0261747.s003.docx]

**Appendix: C (Sample Calculations)**

**(i) Cronbach Alpha (α) calculations**

The sample calculation is shown for quality performance of hospitals (Table 6). Cronbach alpha (α) can be calculated from the relation given in Equation C1.

$\alpha= \frac{\bar{r} \times k}{[1+(k-1)\bar{r}}$ **(C1)**

Where k = no. of items,

$\bar{r}$ = the mean of inter-item correlations,

$\bar{r}$ is computed to be 0.585 and k = 7 for quality performance

So, Cronbach alpha (α) for **quality performance** is computed as:

α = (0.585 × 7) / [1+ (7-1) × 0.585]

Cronbach alpha for quality performance = 0.908

**(ii) Factor Loading calculation**

The calculation of factor loading is shown for item 1 to item 4 of **quality performance** (Table 7). First, the covariance of different items is computed using Equation C2.

$Covariane \left( X_{i} \right)= \frac{\sum_{i=1}^{n} (X_{i}-\bar{X})(Y_{i}-\bar{Y})}{n}$ **(C2)**

Where X_i_ refers to a particular item and Y_i_ denotes the sum of all other items. So, the values of the covariance of items are as follow:

For Item 1 = 0.375, for Item 2 = 0.485, for item 3 = 0.380, for Item 4 = 0.323

The summary of results is given in Table 1C.

**Table 1C. Results for Factor loading for item 1 to 4 of quality performance.**

| **Item** | **Covariance** | **Communalities = 1 - Covariance** | **Factor loading (square root of communalities)** |
| --- | --- | --- | --- |
| Item 1 | 0.375 | 0.625 | 0.791 |
| Item 2 | 0.485 | 0.515 | 0.718 |
| Item 3 | 0.380 | 0.620 | 0.788 |
| Item 4 | 0.323 | 0.677 | 0.823 |
